# Pediatric GI Health Care Professionals’ Perceptions of and Engagement With Psychosocial Providers in Pediatric IBD Care

**DOI:** 10.1097/PG9.0000000000000305

**Published:** 2023-04-18

**Authors:** Jennie G. David, Ellen Sejkora, Hilary K. Michel, Laura Mackner

**Affiliations:** From the * Nationwide Children’s Hospital, Columbus, OH; † Department of Pediatrics, The Ohio State Wexner Medical Center, Columbus, OH; ‡ Dartmouth Health Children’s, Lebanon, NH; § Nationwide Children’s Hospital, Columbus, OH; ∥ Nationwide Children’s Hospital, Columbus, OH; ¶ Department of Pediatrics, The Ohio State Wexner Medical Center, Columbus, OH; # Center for Biobehavioral Health, Nationwide Children’s Hospital, Columbus, OH.

**Keywords:** IBD, multidisciplinary care, psychosocial care, integrated care

## Abstract

Multidisciplinary care is recommended for optimal pediatric inflammatory bowel disease (IBD) care, including psychosocial providers (eg, psychologists). However, health care professionals’ (HCPs) perceptions of and engagement with psychosocial providers in pediatric IBD is lacking.

**Methods:**

Cross-sectional REDCap surveys were completed by HCPs (eg, gastroenterologists) across American ImproveCareNow (ICN) centers. Demographics and self-reported perceptions of and engagement with psychosocial providers were collected. Data were analyzed at participant and site levels through descriptives, frequencies, an independent *t* test, and exploratory analyses of variance.

**Results:**

A total of 101 participants from 52% of ICN sites participated. Participants were 88% gastrointestinal physicians, 49% identifying as female, 94% non-Hispanic, and 76% Caucasian. Of ICN sites, 75% and 94% of sites reported outpatient and inpatient psychosocial care, respectively. Participants referred for various clinical reasons to psychosocial providers (eg, illness adjustment). At the participant level, 92% of HCPs reported psychosocial care was very important and 64% reported their clinical thresholds shifted to engage psychosocial providers earlier in care. Barriers to psychosocial care included limited psychosocial providers (92%), psychosocial providers availability (87%), and IBD patients’ lack of openness to psychosocial care (85%). One-way analyses of variance by HCP length of experiences were not statistically significant on perceived understanding of psychosocial providers or perceived changes in clinical threshold over time.

**Conclusion:**

HCPs overall reported positive perceptions of and frequent engagement with psychosocial providers in pediatric IBD. Limited psychosocial providers and other notable barriers are discussed. Future work should continue interprofessional education of HCPs and trainees and efforts to improve access to psychosocial care in pediatric IBD.

**What Is Known**

- Multidisciplinary care is the standard of care in pediatric inflammatory bowel disease (IBD).
- Psychosocial providers, including psychologists and social workers, are part of multidisciplinary care.

**What Is New**

- GI health care professionals (HCPs) endorsed positive perceptions of psychosocial providers in pediatric IBD care, including endorsing that psychosocial care is important.
- Outpatient and inpatient psychosocial care was identified as available at some IBD centers across the US, with notable need for increased access.
- Barriers to psychosocial care are identified from HCP perspectives, included limited access to psychosocial providers and patients and families lack of openness to psychosocial care.

## INTRODUCTION

Pediatric inflammatory bowel diseases (IBD) are a group of chronic, immune-mediated diseases where multidisciplinary care is considered the standard of care (1–7). Multidisciplinary care for pediatric IBD includes traditional medical roles as well as psychosocial care, including but not limited to pediatric psychologists, social workers, and child life specialists (6,7).

The psychosocial considerations of pediatric IBD, including anxiety, depression, adherence, transition to adult care, and body image (5,6,8), have been documented and clearly impact care for patients with IBD. While psychosocial concerns are known to be impactful in pediatric IBD, it is unknown how health care professionals (HCPs) (eg, gastroenterology/gastrointestinal physicians [GIs], advanced practice nurses [APNs], nurse practitioners [NPs], and physician assistants [PAs]) perceive psychosocial care and providers. HCPs’ perceptions and beliefs have the potential to impact engagement with psychosocial care in pediatric IBD, and therefore may impact patients’ access and outcomes. However, empirical evidence about how pediatric gastroenterology HCPs perceive of and engage with psychosocial providers in pediatric IBD care is lacking. First, we sought to characterize HCPs’ perceptions about and experiences with psychosocial providers in pediatric IBD, and second, describe IBD center-level data related to access to psychosocial services in this population.

## METHODS

A cross-sectional REDCap survey study developed by the authors was disseminated electronically to pediatric GI HCPs (GIs, APNs/NPs/PAs) across American hospitals through the ImproveCareNow (ICN) network, a Learning Health System focused on pediatric IBD (9). The REDCap survey questions related to perceptions of and engagement with psychosocial providers are available as a Supplemental File, Supplemental Digital Content, http://links.lww.com/PG9/A105. At the time of the study, there were 98 American ICN sites. Participants were recruited over a 4-month period beginning in November, 2021, via biweekly emails to the ICN network, posts to ICN’s intranet, and emails to ICN site contacts. Participant-level data collected via self-report included demographics, information on professional role (eg, dedicated time to pediatric IBD), perceptions of psychosocial providers, and engagement/experiences with psychosocial providers.

The survey also collected ICN site-level data related to IBD psychosocial services at their center, such as the type(s) of psychosocial providers available (eg, psychologist) and the settings of these psychosocial providers (eg, outpatient). If >1 participant per site completed the survey, all responses for the site were reviewed for congruency and collapsed into 1 site by the research team using available ICN data (ie, knowledge that a psychologist worked at a given center), resulting in each participating site having only 1 entry in the site-level dataset. ICN site-level data were also extracted from the ICN registry, and included the number of patients diagnosed with IBD actively receiving care at a given site and whose legal guardians have consented to participate in the ICN registry. Active ICN registry values were extracted as of November 1, 2021, which corresponds to the date that participant recruitment began. This study received IRB approval and a waiver of informed consent, although agreement to participate was indicated by survey completion. Data were analyzed at the participant and site level through descriptive statistics, frequencies, an independent *t* test, and exploratory analyses of variance (ANOVAs) using SPSS. Dependent variables of interest were assessed for skewness and transformed accordingly before exploratory ANOVAs to assess potential relationship between length of HCP experience and perceptions toward psychosocial providers.

## RESULTS

A total of 101 participants across 52% (n = 51) of the American ICN sites completed the survey. Participants were primarily GI physicians (88%, n = 86) with 10% (n = 10) NP/APNs and 2% (n = 2) PAs. Two-thirds (66%, n = 67) had worked in pediatric IBD for ≥10 years, with 49% (n = 49) identifying as female, 94% (n = 95) as non-Hispanic, and 76% (n = 75) as Caucasian. Please see Table 1 for overview of participants.

**TABLE 1.** Self-reported HCP participant demographics

	n	%
HCP role		
Pediatric gastroenterologist	86	88%
Advanced practice nurse/nurse practitioner	10	10%
Physician assistant	2	2%
HCP Gender		
Female	49	49%
Male	51	51%
Prefer not to say	1	1%
HCP Ethnicity		
Hispanic	3	3%
Non-Hispanic	95	94%
Prefer not to say	3	3%
HCP Race		
Caucasian	75	76%
Black	2	2%
Asian	13	13%
Multiracial	2	2%
Other	3	3%
Prefer not to say	4	4%
Percentage of clinical work with IBD patients		
<10%	36	36%
25%	29	29%
50%	19	19%
75%	10	10%
100%	7	7%
Duration of time working in IBD (in years)		
<3	2	2%
3 to <5	19	19%
5 to <10	14	14%
10 to <15	21	21%
15 to <20	21	21%
≥20	25	25%

HCP = health care professional; IBD = inflammatory bowel disease.

### Participant-Level Results

In outpatient pediatric IBD care, 19% of respondents reported having joint visits with a psychosocial provider and half (51%) reported that a psychosocial provider could see a patient the same day/same visit when needed. Participants endorsed various reasons to refer to psychosocial providers from a list of generated referral reasons informed by literature on known psychosocial needs in IBD and clinical practice insights (5,6,8) (Table 2) with the 3 most frequent reasons being coping with symptoms (78%), adjustment to diagnosis (77%), and current mental health concerns (76%).

**TABLE 2.** Referral reasons for psychosocial care

Referral reasons	n	%
Coping with symptoms	79	78%
Adjustment to diagnosis	78	77%
Current mental health concerns	77	76%
Adherence concerns	62	61%
Family systems concerns	53	53%
Pain management	51	51%
Historical mental health concerns	50	50%
Self-harm	49	49%
Suicidal ideation	48	48%
Social or school concerns	43	43%
School avoidance	43	43%
Procedural anxiety	40	40%
Engagement challenges	39	39%
Transition readiness	35	35%
Pill swallowing	31	31%
Cognitive/developmental concerns	24	24%
Psychoeducational evaluation	14	14%
Ostomy teaching	6	6%
Other	4	4%

Participants endorsed several potential barriers from a generated list of barriers informed by pediatric psychology literature and clinical insights (5,10,11) to involving psychosocial providers in care in pediatric IBD. The most common barriers endorsed as somewhat to very difficult were lack of/limited psychosocial providers (92%), time/availability of psychosocial providers (87%), lack of openness of the IBD patient to psychosocial care (85%), lack of openness of the patients’ family to psychosocial care (84%), limited/lack of insurance coverage for psychosocial care (78%), and geographical constraints in accessing psychosocial care (ie, limited mental health in an area) (71%).

The vast majority of HCPs (92%) identified psychosocial care as very important and most (80%) reported that they felt very comfortable working with psychosocial providers. Two-thirds (64%) reported that their own clinical thresholds had changed to engage psychosocial care earlier in care over their own years of practices and 64% reported that they often/always talk with medical trainees about the role of psychosocial care in pediatric IBD. Participants endorsed learning about psychosocial care in pediatric IBD in various ways, including noticing the impact on patients with psychosocial care (54%), asking a psychosocial colleague (51%), observing a psychosocial colleague providing care (49%), attending a presentation in their department (30%), and independently seeking out a talk/presentation (21%).

Participants reported perceptions that approximately 66% of outpatient pediatric IBD visits would benefit from psychosocial care during visits, thought estimated that only 26% currently had access to a psychosocial provider during outpatient pediatric IBD visits. The most commonly reported factors that influenced the likelihood of a HCP referring a patient to psychosocial care included patients already being connected with a psychosocial provider (45% of respondents) and the degree or impact of a patient’s psychosocial needs (62% of respondents).

### ICN Site-Level Results

**FIGURE 1.**
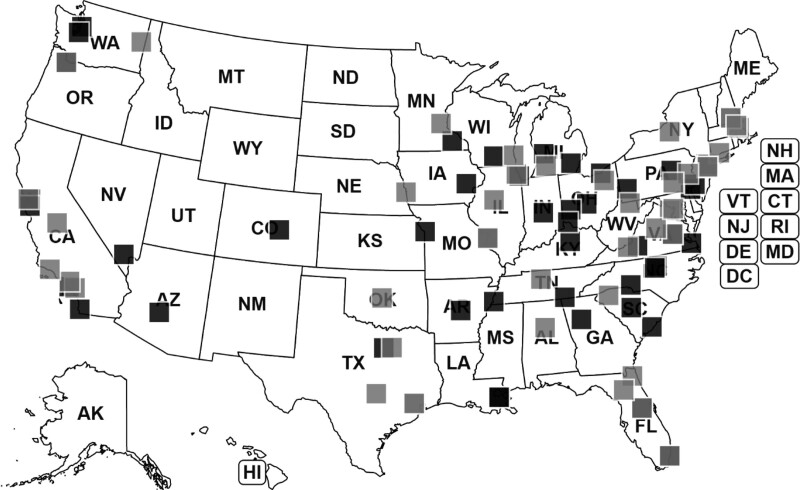
Geographical distribution of ImproveCareNow (ICN) centers participating and nonparticipating in this study. Participating ICN centers □ Nonparticipating ICN centers

Participating sites represented all geographical areas in the US; participating and nonparticipating sites were similarly distributed across the country (Fig. 1). Three-quarters of ICN sites endorsed access to psychosocial providers for outpatient pediatric IBD care and 94% reported access to psychosocial providers for inpatient IBD care. Table 3 summarizes geographical and patient population descriptive data for participating and nonparticipating ICN sites. Participating ICN sites had significantly more patients than nonparticipating sites (M = 371.8, SD = 313.6 versus M = 252.2, SD = 164.5, respectively), *t*(88) = 2.16, *P* = 0.03.

**TABLE 3.** Site-level data for United States ICN centers participating versus those not participating in this study

Variable	Participating sites		Non-participating sites	
IBD patient population per site from active ICN registry population (data as of January 11, 2021)	n	M (SD)	n	M (SD)
	51	371.8 (313.6)	39	252.2 (164.5)
Geographical region	n	%	n	%
New England	2	4%	5	11%
Northeast	8	16%	5	11%
East Midwest	10	20%	2	5%
Atlantic coast	8	16%	6	14%
Southeast	5	10%	6	14%
West Midwest	6	12%	8	18%
South central	3	6%	5	11%
Pacific northwest	9	18%	7	16%

IBD = inflammatory bowel disease; ICN = ImproveCareNow; M = mean.

### Exploratory ANOVAs

Exploratory ANOVAs assessed HCPs’ perceptions toward psychosocial providers based on the length of HCP experience (grouped into 3 levels of <5 years [21%], 5 to <15 years [59%], and ≥15 years [21%]). Dependent variables of interest, (1) participant’s perceived understanding of psychosocial providers’ role (on a 5-point Likert scale from “do not understand at all” to “understand completely,” with 3% of participants endorsing not understanding and 81% endorsing understanding) and (2) participant perception of whether their clinical threshold for engaging psychosocial providers had changed throughout practice, were assessed for skewness. One dependent variable (1) was found to be negatively skewed and was logarithmically transformed to correct for skewness. A 1-way ANOVA did not identify statistically significant differences on perceived understanding of psychosocial providers’ care by duration of HCP experience, *F*(2, 27) = 1.81, *P* = 0.17. Another 1-way ANOVA also did not identify statistically significant differences on perceived changes in clinical threshold over time by duration of HCP experience, *F*(2, 85) = 1.2, *P* = 0.33.

## DISCUSSION

Psychosocial care in IBD is critical to patients’ care experiences and can support various clinical needs throughout the treatment (4,5,8,12). The current study sought to describe HCPs’ perceptions and experiences with psychosocial providers in pediatric IBD, as well as ICN center-level data related to access of psychosocial providers. This study identified that HCPs had largely positive perceptions of psychosocial care in pediatric IBD, with most HCPs describing psychosocial care as very important to their patients. Most HCPs also reported feeling that they understand the value of psychosocial care in pediatric IBD, and they feel very comfortable working with psychosocial providers. This work also identified limited overall availability and access to psychosocial providers at the ICN-center level. Most HCPs identified that their clinical thresholds had evolved throughout their practice such that they were increasingly likely to engage psychosocial providers earlier for their pediatric IBD patients. Despite HCPs self-reporting a personal change, this change was not statistically significant per exploratory ANOVAS and warrants further examination with a larger sample size to better understand change in provider attitudes and beliefs about psychosocial care over time. It is likely that familiarity increases over time through the informal and transdisciplinary education experiences identified by the HCPs in this study. This highlights an important interprofessional opportunity for psychosocial providers to partner and build relationships with HCPs in order to model psychosocial care and identify occasions for cotreatment. Participants in this study also reported often educating their medical trainees about the role of psychosocial care in pediatric IBD, which has been identified as an educational area of need based on work showing that 64% of GI fellows feeling interested to support psychosocial needs of their patients, while only 25% endorsing feeling competency to do so (13).

Unfortunately, there are numerous perceived barriers to integrated psychosocial care in pediatric IBD. Importantly, the 2 most common barriers endorsed—lack of psychosocial providers and limited availability of psychosocial providers—highlights an urgent need to increase psychosocial positions in IBD, including hiring more psychosocial providers with more time dedication to IBD. While the majority of ICN sites endorsed having access to psychosocial providers in outpatient pediatric IBD care and nearly all sites reported some access to psychosocial providers for inpatient pediatric IBD care, these findings demonstrate an ongoing notable need for increased number of psychosocial providers and time dedication/full-time equivalent status in IBD. Increasing the psychosocial presence in pediatric IBD may be supported by physician champions advocating within their medical departments and the associated departments that house psychosocial providers (eg, Psychology and Psychiatry), and by educating department leaders about the nuanced and integral work that psychosocial providers can provide in pediatric IBD. Of note, the contributions of psychosocial providers may also reduce/manage the workload of HCPs; examples include psychosocial providers managing school-related accommodations, addressing adherence, and providing care related to disorders of gut-brain interaction. It is also important to highlight that the interest in and appreciation of psychosocial providers in IBD may be different than an institution’s ability to hire and support psychosocial providers. While financial investment is necessary, HCPs and institutional leadership should familiarize themselves with the growing body of literature demonstrating how incorporating value-based care, including psychosocial care can, can improve patient outcomes and contribute to cost savings (14–16).

Our findings suggest that HCPs generally endorsed an awareness of the broad topics that psychosocial providers have expertise in and refer IBD patients to psychosocial care for various needs, which is consistent with the extant literature (5,8). Participants also endorsed that patient and family lack of openness to psychosocial care were key perceived barriers to integrated care in IBD. This may be related to the stigma associated with receiving mental health care in pediatrics (17,18), misperceptions of indications for involvement of psychosocial providers (ie, psychosocial providers only being engaged when there is a significant concern), previous negative experiences with mental health discussions, unfamiliarity with what IBD psychosocial providers can do (ie, more than anxiety and depression screening), and lack of rapport/familiarity with IBD psychosocial providers at their center. Although the authors are hopeful that increasing awareness and discussion about mental health in literature and clinical care will positively contribute to patients and families being open to psychosocial care in IBD, there are also notable and pressing action steps to take from this finding.

As GI HCPs likely have the most rapport with the patient/family of the IBD team, HCPs discussing and modeling how psychosocial care in IBD is the standard of care may help to shift patient and family perspectives; for example, a HCP telling a family, “We know that young folks with IBD are more likely to experience anxiety, mood concerns, and can also have tough experiences with other things like fatigue and body image. We work closely with our psychologist/social worker and I’d love for them to join us today and see how they can support you/your child,” may help to share the rationale and set the expectation for multidisciplinary care. Integrated multidisciplinary IBD care may help to reduce perceived stigma of meeting with psychosocial providers. For example, rather than patients needing to opt-in to a visit with psychosocial providers, they could be given an opportunity to opt-out of the standard visit model and instead request not to be seen.

In the experiences of the authors (J.G.D. and H.K.M.), the IBD psychologist joining the visit at the same time as the HCP allows for the psychologist to be a friendly face, allows the HCP to use inclusive language that incorporates the psychologist (eg, “Tell us about how you are feeling”), gives the HCP the option to defer topics to the psychologist (eg, “I’m going to let you have more time with Dr. David to talk about difficulties you’ve had giving your injections”), and allows the psychologist to engage in anticipatory guidance, psychoeducation, assessment, and intervention. As an example from the psychologist’s perspective (J.G.D.), a young adult with IBD who identified as nonbinary expressed concerns related to their body size and interest in reducing their weight status. Through collaborative conversation between the psychologist, HCP, and patient, body image complexities were identified to be unrelated to medical consideration, although highly related to the patient’s perception of their body/body image. Consequently, the psychologist stayed with the patient after the HCP left the room to provide support and interventions related to body image in IBD. As an example from the GI physician’s perspective (H.K.M.), during a recent visit with a teenage boy with ulcerative colitis, severe fatigue was identified as a primary concern for his visit, one common to patients with IBD. After reviewing the many potential causes of fatigue with the patient and his family, the GI HCP and psychologist conducted a sleep history and identified that the patient had erratic sleep and wake times, took long naps after school, and left the television on in his room at night to help him fall to sleep. Identifying sleep hygiene as an area for improvement, the GI psychologist was able to stay with the patient and his family to provide education and set goals, allowing the GI HCP to complete her portion of the visit and move on to the next patient.

Previous work related to pediatric primary care HCPs’ perceptions of mental health identified similar patterns of discrepancy between perceived need and reduced access to integrated care (19). Similarly, training and knowledge of mental health needs among primary care HCPs and HCPs’ stigma related to mental health have been proposed as barriers to integrated care within primary care clinics (19). Imfeld and colleagues found that variation in mental health training and familiarity impacted pediatricians’ perceptions and practice (ie, a pediatrician with more exposure to mental health concerns was more likely to feel comfortable discussing these concerns). Mental health care management in primary care was also found to be related to the clinic itself (eg, having psychologists at the clinic), with perceptions of mental health care by pediatricians influenced by the clinic set-up/culture. This previous work also highlights the importance of providers spending time reflecting on their own perceptions of mental health and stigma to work toward minimizing the impact of stigma/personal perceptions on clinical care. Continued stigma at the level of the provider and the patient highlights another area where informal education can help HCPs feel more comfortable involving psychosocial providers in care; this barrier would likely benefit from HCP colleagues having coaching/modeling from psychosocial providers and how to present psychosocial services to patients and families.

Given the importance of understanding the whole patient and family and in the context of knowledge that pediatric patients with IBD are often impacted by psychosocial needs, HCP medical training would strongly benefit from systematic education and exposure to psychosocial providers; this may also arm HCPs with specialized language or strategies to improve their care delivery as well, such as how to assess adherence, supporting developmentally-appropriate understanding of IBD and treatments, and insights in how to have a difficult conversation.

### Limitations

Some limitations may have influenced the results of this survey. While geographical data from this study identified similar geographical distributions between ICN sites who did and did not participate, potential differences related to regional perceptions/culture regarding mental health stigma may not be fully represented in the present study. Participating sites had statistically significantly more patients than nonparticipating sites, which likely reflects that larger ICN sites participated in this study. This overrepresentation of sites with larger patient populations is a limitation to be mindful in interpreting results for ICN sites with smaller IBD population. Further, participating sites may also overrepresent integration of psychosocial providers compared to nonparticipating sites, which may have influenced results evaluated at the participant-level. Lastly, pediatric IBD centers who participate in ICN may experience more exposure and awareness to the role of psychosocial care, which may in turn impact perceptions of and engagement with psychosocial providers, potentially limiting the generalizability of this work to non-ICN sites. Per data shared by ICN leadership as of this article submission, ICN represents 55% of practicing pediatric GIs and 60% of pediatric GI practices (unpublished data).

### Future Directions

Although this study focused on GI HCPs’ perceptions of and experiences with psychosocial providers in pediatric IBD care, it is also important to highlight the evolving landscape of pediatric IBD (eg, Very Early Onset IBD, perioperative medical decision-making, transitioning of care) and the psychosocial care these needs may benefit from. Future research should seek to understand how to have equitable access to integrated IBD care with psychosocial providers to pediatric IBD patients throughout development and the disease process. Future work should also seek to understand what, if any, aspects of perceptions of and engagement with psychosocial providers in pediatric IBD evolve over time and if this relates to changes in development or the disease process.

While pediatric GI HCPs endorsed highly positive perceptions of psychosocial providers in pediatric IBD and also reported being very receptive to multidisciplinary care, limited access to psychosocial providers continues to be a significant barrier in integrated IBD care. Medical-psychosocial education partnerships, both in pediatric GI fellowship training as well as among trained HCPs may help reduce stigma and build understanding of the vital role that psychosocial providers play in the management of pediatric IBD. National, systematic efforts throughout ICN partner sites are likely needed to equitably improve psychosocial care access in multidisciplinary for all patients with pediatric IBD. There are numerous and urgent clinical applications of these findings in improving care now for pediatric IBD patients and their families.

## Supplementary Material
