# Supplementary material for: Pediatric GI Health Care Professionals’ Perceptions of and Engagement With Psychosocial Providers in Pediatric IBD Care
Source: JPGN Rep. 2023 Apr 18;4(2):e305. doi: 10.1097/PG9.0000000000000305 (PMC10187836; doi:10.1097/PG9.0000000000000305)
Supplement: Supplementary file 1 [file pg9-4-e305-s001.pdf]

Experience Questions

Please tell us about your experiences with psychosocial providers (psychologists and social workers) in the OUTPATIENT gastroenterology clinic setting:

11. Do you have access to psychosocial providers at your center in the OUTPATIENT gastroenterology clinic setting? (if no, go to question 20)

- ◯ No
- ◯ Yes
- ◯ Unsure

Please skip to question #20.

12. Which of the following OUTPATIENT gastroenterology clinic visit types are psychosocial providers available at? (Check all that apply):

- ☐ New patient visits
- ☐ Return patient visits
- ☐ Unsure

13. Which types of psychosocial providers to you have access to in the OUTPATIENT gastroenterology clinic setting? (Check all that apply):

- ☐ Pediatric psychologists
- ☐ Social workers
- ☐ Master's level therapists
- ☐ Neuropsychologists
- ☐ Child life specialist
- ☐ Other

If you selected Other, please tell us what type of provider:

____

14. What is the psychosocial providers' dedication to IBD patients in the OUTPATIENT setting (If multiple providers, please select the maximum dedication)?

- ◯ Full-time
- ◯ Part-time
- ◯ Unsure

15. In what ways are psychosocial providers involved in the care of IBD patients at your center in the OUTPATIENT setting? (Check all that apply):

- ☐ Joint visits with medical provider
- ☐ Psychosocial provider sees patient after medical provider, whether or not a concern is identified
- ☐ Psychosocial provider sees patient after medical provider, only if a concern is identified
- ☐ Patients care seen at separately scheduled visits with a psychosocial provider
- ☐ Other

16. Do your psychosocial providers see patients via telehealth? If yes, in which formats? (Check all that apply):

- ☐ N/A, psychosocial providers do not have ability to see patients via telehealth
- ☐ Telephone
- ☐ Video visits
- ☐ Unsure

17. If a clear psychosocial need arose, how soon would a psychosocial provider see the IBD patient in the OUTPATIENT setting?

- ◯ Same visit/same day
- ◯ A separate visit/within one week
- ◯ A separate visit/within one month
- ◯ Unsure

18. Approximately what percentage of your IBD patients currently see a psychosocial provider during their OUTPATIENT GI visits?

50 100

(Place a mark on the scale above)

19. Approximately what percentage of your IBD patients would benefit from seeing a psychosocial provider during their OUTPATIENT GI visits?

50

(Place a mark on the scale above)

20. Approximately what percentage of your IBD patients currently see a psychosocial provider after or in addition to their outpatient GI visits?

50

(Place a mark on the scale above)

21. Do you ever refer your IBD patients to psychosocial providers OUTSIDE of your center?

- ◯ No
- ◯ Yes
- ◯ Unsure

22. For what reasons do you refer patients to see psychosocial providers? (Check all that apply)

- ☐ Procedural anxiety
- ☐ Pill swallowing
- ☐ Poor adherence/disease management
- ☐ Limited engagement with medical team
- ☐ Difficulty adjusting to diagnosis
- ☐ Coping with symptoms
- ☐ Pain management (including biofeedback)
- ☐ Transition readiness
- ☐ Cognitive/developmental concerns
- ☐ Social or school challenges
- ☐ School avoidance
- ☐ Ostomy Teaching
- ☐ Psychoeducational testing (i.e., learning concerns)
- ☐ Current mental health concerns (anxiety, depression)
- ☐ History of mental health concerns
- ☐ Self-harm
- ☐ Suicidal ideation
- ☐ Family dynamic issues
- ☐ Other

If you selected Other, please tell us more:

____

Please tell us about your experiences with psychosocial providers in the INPATIENT medical setting.

23. Do you have access to INPATIENT psychosocial consults at your center?

- ◯ No
- ◯ Yes
- ◯ Unsure

Please skip to question #25.

24. If yes to question 23, which types of INPATIENT psychosocial providers to you have access to? (Check all that apply):

- ☐ Pediatric psychologists
- ☐ Social workers
- ☐ Master's level therapists
- ☐ Neuropsychologists
- ☐ Child life specialist
- ☐ Other

If you selected Other, please tell us what type of provider:

____

The following question addresses barriers and facilitators to providing psychosocial care for IBD patients at your center:

25. How feasible is it for you to refer IBD patients to knowledgeable psychosocial providers who work at your institution?

- ◯ Not at all feasible
- ◯ Somewhat not feasible
- ◯ Neutral
- ◯ Somewhat feasible
- ◯ Very feasible
- ◯ Not applicable

26. How difficult do the following make it for your patients to receive psychosocial care in any setting

	Not at all difficult	Somewhat difficult	Very difficult
Clinic time/flow	◯	◯	◯
Clinic space/rooming	◯	◯	◯
Number of psychosocial providers	◯	◯	◯
Time/availability of psychosocial providers	◯	◯	◯
IBD experience amongst psychosocial providers	◯	◯	◯
Ability to reach psychosocial providers	◯	◯	◯
Ease of referral process	◯	◯	◯
Geographic constraints	◯	◯	◯
Insurance coverage	◯	◯	◯
Patient's openness to psychosocial providers	◯	◯	◯
Family's openness to psychosocial providers	◯	◯	◯

27. How do the following impact your likelihood of referring a patient to a psychosocial provider?

	Less likely	No change	More likely
Whether a patient is already linked to outside support	◯	◯	◯
Impact of psychosocial concerns on the patient/family (e.g., impact on child's functioning)	◯	◯	◯

28. Are there any other factors that impact your likelihood of referring a patient to a psychosocial provider?

◯ No ◯ Yes

If Yes, please tell us more:

____

These questions address your attitudes and feelings toward psychosocial care for children with IBD:

29. How important do you think psychosocial care is for pediatric and adolescent patients with IBD?

- ◯ Not at all important
- ◯ Somewhat unimportant
- ◯ Neutral
- ◯ Somewhat important
- ◯ Very important

30. How helpful do you feel psychosocial providers are with management of your IBD patients?

- ◯ Not at all helpful
- ◯ Somewhat unhelpful
- ◯ Neutral
- ◯ Somewhat helpful
- ◯ Very helpful

31. How comfortable are you with working with psychosocial providers in care of your IBD patients?

- ◯ Not at all comfortable
- ◯ Somewhat uncomfortable
- ◯ Neutral
- ◯ Somewhat comfortable
- ◯ Very comfortable

32. Since you began seeing IBD patients, how has your threshold for requesting involvement of a psychosocial provider changed?

- ◯ My threshold has not changed
- ◯ I tend to reserve referrals for only very high needs patients
- ◯ I tend to request psychosocial providers more for prevention or earlier in care
- ◯ Other

If you selected Other, please tell us more:

____

33. How well do you feel you understand what psychosocial providers do with your IBD patients?

- ◯ Do not understand at all
- ◯ Somewhat do not understand
- ◯ Uncertain
- ◯ Somewhat understand
- ◯ Understand completely

34. How did you learn about what psychosocial providers do in the care of pediatric patients with IBD? (Check all that apply)

- ☐ A presentation was given in my department
- ☐ I sought out a presentation/talk on this topic
- ☐ I asked my psychosocial colleagues
- ☐ I asked my physician/medical colleagues
- ☐ I observed what my psychosocial colleagues did
- ☐ I noticed the impact on my IBD patients who worked with psychosocial providers
- ☐ Other

If you selected Other, tell us more:

____

35. How often do you talk with your medical trainees (e.g., medical students, residents, fellows) about the role of psychosocial care in pediatric IBD?

- ◯ Never
- ◯ Rarely
- ◯ Sometimes
- ◯ Often
- ◯ Always
- ◯ N/A - there are no trainees at my center

36. What other aspects of working with psychosocial providers in caring for IBD patients are important to consider? (e.g., successes, frustrations, challenges, future opportunities)? ____

Thank you for participating in the survey - your participation is greatly appreciated!
